# The U.S. President’s Malaria Initiative’s Support for Improving the Quality of Malaria Case Management Services: Fifteen Years of Progress and Learning

**DOI:** 10.4269/ajtmh.23-0207

**Published:** 2023-11-27

**Authors:** Lawrence M. Barat, Nicole Whitehurst, Meera Venkatesan, Kim Connolly, Emmanuel Yamo, Paul Psychas, Yves-Marie Bernard

**Affiliations:** ^1^ PMI Impact Malaria Project, Population Services International, Washington, District of Columbia;; ^2^ U.S. President’s Malaria Initiative, United States Agency for International Development, Washington, District of Columbia;; ^3^ PMI Impact Malaria Project, Medical Care Development—Global Health, Silver Spring, Maryland;; ^4^ U.S. President’s Malaria Initiative, Centers for Disease Control and Prevention, Lusaka, Zambia

## Abstract

Since its launch in 2005, the U.S. President’s Malaria Initiative’s (PMI) investment in malaria case management has evolved based on lessons learned from its support to countries. An initial focus on updating malaria treatment policies to adopt artemisinin-based combination therapies achieved limited success, in part because of the poor quality of diagnostic and treatment services in targeted countries. In response, the PMI supported the development, refinement, and expansion of Outreach Training and Supportive Supervision (OTSS), a quality improvement approach that combines structured, competency-based supervision with corrective measures, including on-the-job training, coaching, troubleshooting, action planning, and timely follow-up. With 15 years of experience, the OTSS approach has been adopted by more than a dozen countries, and its effectiveness in improving the quality of malaria case management services has been documented. Through the PMI Impact Malaria Project, launched in 2018, the OTSS approach was expanded beyond case management of uncomplicated malaria to support quality improvement of inpatient management of severe malaria and malaria in pregnancy services delivered through antenatal care clinics. The OTSS platform also enabled targeted countries to respond rapidly to the COVID-19 pandemic by adding modules related to clinical management and laboratory diagnosis of suspected cases. The OTSS approach has been established as an effective approach to improve the quality of clinical malaria services and can be expanded to cover other health priorities. Further innovations to improve the quality of inpatient and community-based services, and further integration and institutionalization of OTSS into country health systems are needed.

## U.S. PRESIDENT’S MALARIA INITIATIVE’S EARLY SUPPORT FOR MALARIA SERVICE DELIVERY

After decades of inattention by the global community and affected countries, the launch of Roll Back Malaria in 1998 and the Abuja Declaration in 2001 heralded a renewed interest in reducing death and disease from malaria.^1^ Newer, more effective tools, including insecticide-treated bed nets (ITNs), artemisinin-based combination therapies (ACTs), and malaria rapid diagnostic tests (mRDTs) offered hope that the burden of malaria could be greatly reduced. The launch of the Global Fund for AIDS, Tuberculosis, and Malaria (Global Fund) in 2003 and the U.S. President’s Malaria Initiative (PMI) in 2005 mobilized new, substantial funding for malaria control for the first time in many decades. This infusion of funding enabled malaria-affected countries to scale up these new tools.

The PMI focused early investments on scaling up preventive strategies, such as ITNs and indoor residual spraying, to demonstrate that investments in malaria control could yield rapid results, making the case for expanded funding.^2^ This focus on achieving early wins led the PMI to target its early support for malaria case management to assisting national malaria programs (NMPs) to implement the WHO’s 2001 guidance to adopt ACTs as first-line treatment of uncomplicated malaria and to introduce mRDTs.^3^ Focusing on replacing failing malaria treatments, such as chloroquine, with ACTs was viewed as the most efficient means of improving malaria outcomes, particularly malaria mortality, in the short term, with the limited resources available at the time. The PMI annual report for 2007^2^ listed its key accomplishments for malaria case management as procurement of ACTs and mRDTs, and training health workers in their use.

However, there were additional barriers to accessing high-quality malaria services. For decades, the diagnosis of a large majority of malaria cases in sub-Saharan Africa had been based solely on clinical symptoms (fever) and physical findings. Common practice at the time was that any child with a history of fever would be treated for malaria when diagnostic testing was not available, which was most often the case. This guidance was based on ample evidence that large percentages of those presenting to health facilities with fever had malaria parasitemia.^4^ In addition, the widespread availability of cheap and safe malaria treatments, such as chloroquine, coupled with a lack of diagnostic testing capacity in most front-line facilities provided a strong rationale for so-called “clinical” diagnosis and treatment.

Despite the PMI’s contributions to global efforts to scale up ACTs and mRDTs, the 2010 world malaria report^5^ estimated that less than 20% of all suspected malaria cases in sub-Saharan Africa were tested, and only 65% of all malaria cases received treatment. Just 11 countries in sub-Saharan Africa had sufficient quantities of ACTs to meet their needs, and more than half of the countries had less than half the ACTs they required.

## UPDATED WHO GUIDANCE, THE LANTOS-HYDE ACT, AND NEW OPPORTUNITIES TO STRENGTHEN MALARIA SERVICE DELIVERY

This lack of progress in malaria case management stimulated the WHO to update its diagnostic and treatment guidelines in 2009 and 2010, respectively.^6^^,^^7^ The new treatment guidance stated, “Parasitological confirmation of the diagnosis of malaria provided by high-quality microscopy or, where this is not available, by RDTs is recommended for all suspected cases of malaria.”^7^ The higher cost of ACTs, the growing availability of low-cost mRDTs, and decreasing malaria prevalence in countries all were factors leading to this change in guidance.

The PMI was well positioned to support countries to respond to these updated guidelines after the passage of the Lantos-Hyde U.S. Global Leadership against HIV/AIDS, Tuberculosis, and Malaria Reauthorization Act of 2008, under which overall annual funding for the PMI was expanded from $30 million in 2005 to $500 million in 2010, along with expanded support from three countries to a total of 15.^8^ The PMI also had launched the Improving Malaria Diagnostics (IMaD) Project in 2007, the main objective of which was to improve the quality of malaria diagnostic testing and increase effective treatment at health facilities.

The IMaD Project, the first PMI-funded global project to tackle quality improvement (QI) for malaria, faced a difficult environment. Diagnostic testing for malaria, particularly malaria microscopy, was largely unavailable. Laboratory assessments conducted by IMaD focus countries revealed that guidelines for malaria diagnosis were not up to date, refresher trainings were rarely conducted, and supervision/QI programs were not operating.^9^ These challenges were common throughout sub-Saharan Africa at the time.^10^ In addition, many clinicians did not trust malaria microscopy results, which were often of poor quality, and they were skeptical of mRDTs, which were just being introduced to health facilities. They most often relied on their clinical judgment alone.^11^^,^^12^ If clinicians suspected malaria, they commonly would ignore a negative test result and prescribe malaria treatment. If clinical practice was to change, malaria diagnostic testing quality would have to improve, and clinicians would have to trust and abide by the results.

## OUTREACH TRAINING AND SUPPORTIVE SUPERVISION

With increased funding and expansion to 15 countries, the PMI expanded its country support for malaria case management—including procurement of ACTs, mRDTs, and microscopy supplies—and for refresher training of clinical and laboratory staff. The PMI also supported countries in conducting therapeutic efficacy studies to monitor for the emergence of drug resistance. Aligned with the PMI strategy, a number of countries developed tailored approaches to support strengthening of malaria case management.

Through the IMaD Project and two central projects that would follow, the MalariaCare Project (2012–2017) and the PMI Impact Malaria Project (2018–2024), the PMI initiated long-term investments to develop and refine an innovative QI approach for clinical and laboratory services at front-line health facilities. This approach was informed by lessons learned from past unsuccessful approaches, which focused primarily on classroom refresher training and cross-checking of blood slides at reference laboratories.^13^ The IMaD team adopted global best practices for improving provider performance by incorporating effective methods for adult learning, including on-the-job training, coaching, troubleshooting, action planning, and follow-up, to address deficiencies identified during supervision. The effectiveness of these adult learning methods are well documented.^14^^,^^15^

**Figure 1.**
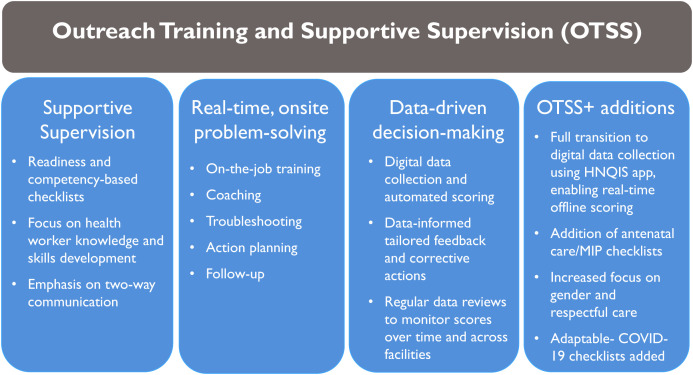
The OTSS quality improvement approach and its key components. HNQIS = Health Network Quality Improvement System; MIP = malaria in pregnancy; OTSS = Outreach Training and Supportive Supervision.

The resulting model was Outreach Training and Supportive Supervision (OTSS), which built on existing country supervision programs while reorienting the approach from inventory and inspection to development and improvement of health worker competencies (Figure 1). Central to the OTSS model was the promotion of a functional, collegial supervisor–supervisee relationship based on two-way communication, respect, and teamwork that emphasized shared responsibilities for making continuous improvements in health worker performance and resolving operational bottlenecks.

The OTSS model was first piloted in Ghana in 2009 in collaboration with the Public Health and Reference Laboratory, which had previously piloted a system in 2004 for monitoring the accuracy of malaria-related laboratory tests using a network of laboratory supervisors. The 2004 pilot showed it could achieve coverage of peripheral health laboratories and staff, improve testing accuracy, and build health worker morale.^16^ The IMaD Project incorporated elements of this external quality assurance program into its OTSS model.

The IMaD Project expanded on Ghana’s existing approach by incorporating clinical supervisors, which acknowledged the complementary roles of laboratory and clinical staff in malaria diagnosis and treatment. Locally adapted, standard, paper-based checklists were introduced to assess provider practices and facility readiness to deliver malaria services (Supplemental 1). Supervisors implementing the OTSS model used a standard method to assess the readiness of health facilities to diagnose and manage appropriately patients presenting with fever, and to assess the competency of health workers in performing key tasks. Laboratory competencies were assessed by observing laboratory staff preparation of malaria blood films, performance of mRDTs, and interpretation of test results. Clinical supervisors observed providers’ abilities to assess the patient and adhere to national diagnostic testing and treatment guidelines. The checklists broke down routine procedures comprehensively into trackable steps, enabling supervisors to determine objectively whether all steps were completed and to identify areas requiring support.

Supervisor training focused both on building technical knowledge and skills, and on instilling good supervisory practices based on supportive supervision principles. Laboratory supervisors received refresher training in malaria microscopy, and clinical supervisors, on clinical case management. Training also was provided on how to provide supportive feedback through coaching, troubleshooting, and problem solving. If, during an observation, a supervisor noted incorrect or unsafe procedures, or identified poorly functioning equipment or substandard supplies, they were trained to intervene after the observation and coach the health worker on the correct procedures and to troubleshoot problems in a respectful manner.

Laboratory supervisors also cross-checked 10 clinical blood slides by microscopy during each OTSS visit. In the event of discordant results, the supervisor took corrective action onsite, including reviewing the discordant slides with the laboratory worker or coaching the laboratory worker on how to prepare Giemsa stain properly. Unlike previous approaches, when slides were sent to reference laboratories for cross-checking, the OTSS approach provided opportunities to identify, correct, and reteach malaria microscopy skills onsite during the OTSS visit. When possible, NMPs also integrated proficiency testing schemes into OTSS visits by hand-carrying sets of well-characterized slides to assess the performance of laboratories in providing accurate results.^13^ Proficiency testing could only be conducted at a small number of facilities because of the limited number of blood slides available.

Building off the 2009 Ghana pilot, supervisors in countries adopting the OTSS model were drawn from national, regional, and district-level Ministry of Health (MOH) staff to facilitate the integration and sustainability of this QI approach. National malaria programs recruited clinical supervisors primarily from district health offices or referral hospitals, whereas laboratory supervisors were drawn from referral hospitals or reference laboratories. Having skilled supervisors at the district level was necessary if this approach was to be scaled up.

If performed efficiently, an OTSS visit generally took one half to a full day to complete, depending on clinic size. After the assessment, supervisors met with health facility staff to discuss results, develop facility-wide action plans with specific benchmarks, and provide on-the-job training and coaching. If essential supplies were lacking at a health facility, the supervisor could liaise with district or regional health staff to address stock outages.

The OTSS approach did not take health facility workers away from their posts, but allowed them to be assessed in their own work environment. It also did not cause major disruptions to health service delivery during the visit. Supervisors also could identify stock outages of commodities, poorly functioning equipment, or lack of clean water and electricity. Using the information collected, OTSS supervisors targeted on-the-job training to areas of weakness, and reported deficiencies that required higher level actions, none of which could occur in a traditional classroom training approach.

Although the OTSS model formed the foundation of the PMI and the IMaD Project’s QI approach, it was only part of a more comprehensive package that included supporting countries to update their policies on the diagnosis and treatment of malaria and targeted classroom training. For instance, malaria diagnostic refresher training was used to build the skills of supervisors, and sometimes frontline staff, leading to the identification of new high-performing supervisor candidates.^17^ Additional support included developing internal quality assurance procedures, and revising and distributing national guidelines documents, standard operating procedures, and job aids. The IMaD Project also initiated plans to support country development of slide banks for training and proficiency testing schemes, and coordinated implementation efforts with partners supporting supply chain management strengthening and social behavior change to improve the availability of essential medicines and commodities, and to strengthen efforts to improve care seeking and adherence to treatment.

By the end of the IMaD Project, the OTSS approach had been adopted by eight PMI-supported countries (Angola, Benin, the Democratic Republic of the Congo, Ghana, Liberia, Malawi, Mali, and Zambia).^17^ To assess the effect of the OTSS model on laboratory and clinical practices after more than 3 years of implementation, the IMaD Project commissioned a cross-sectional survey in 2012 of a random sample of health facilities in Benin and Ghana.^18^ The results of this survey demonstrated that 74% and 88% of microscopy results reviewed in Benin and Ghana, respectively, were accurate, and that more than 80% of all patients who tested positive received malaria treatment. That study^18^ also documented that one third of those with a negative test result also were treated for malaria. The lack of baseline data or a control group, and the limited scale-up of the OTSS model at that time prevented attribution of these results to the implementation of the OTSS approach specifically. A subsequent analysis of data from four rounds of OTSS in Zambia showed significant increases over baseline in the average scores for blood film preparation, blood film staining and reading, and mRDT performance by 14.7%, 14.0%, and 14.3%, respectively.^19^ Improvement of average scores from baseline also was documented for clinical management of fever (+7.3%) and prescriber adherence to negative test results (+7.2%).

## OTSS EXPANSION AND INNOVATION

The close-out of the IMaD Project and launch of the follow-on MalariaCare Project in 2012 provided an opportunity to refine and expand the OTSS model further as a more comprehensive approach to QI, focusing on the development of clinical and laboratory staff. To improve management of children with fever, the OTSS clinical checklist was revised to assess more comprehensively clinicians’ competencies in history taking, physical examination, assessment for danger signs, and identification of nonmalaria infections.^20^ The laboratory checklist was streamlined based on country feedback that it was cost and labor-intensive. MalariaCare also expanded the OTSS model to three additional countries (Kenya, Mozambique, and Tanzania) and made progress in increasing geographic coverage in Zambia, Malawi, and Ghana.

During the IMaD Project and the early years of MalariaCare, paper-based checklists made it challenging to document and disseminate OTSS findings. Although supervisors could intervene and support health workers during the visit, the data collected during these visits only became available to national and subnational health authorities months after supervisors completed their visits. Manual data entry was slowed by poor readability of the completed forms, missing entries, and lost or damaged forms, and typically took as long as 6 months for the final data analysis product to be available.^21^ This made it difficult for supervisors to follow-up or review past findings effectively before revisiting the health facility. These delays also hampered identification of broader systemic issues that could not be addressed in a timely manner.

In 2015, MalariaCare identified an open-source, District Health Information Software 2 (DHIS2)-based electronic data collection tool developed initially to monitor private-sector providers in the Greater Mekong subregion.^22^ This electronic data system (EDS) tool was adapted to support data collection for OTSS using tablet computers. Data were uploaded to a website where NMPs and local health authorities could visualize and track results on dashboards. In the five countries that piloted the EDS tool (Kenya, Mali, Mozambique, Tanzania, and Zambia), data completeness improved from 23% with paper-based forms to 95% with an EDS for clinical observation, and improved from 17% to 70% for microscopy observation.^20^^,^^22^ The average time for data to be analyzed and available decreased from 154 days to 29 days with EDS use. This equipped NMPs and their stakeholders with timely, accurate, and complete data to guide their program planning.

Despite the successes achieved through support by the IMaD Project and MalariaCare, a common critique by some observers was that OTSS and the broader QI package were donor-funded vertical interventions that lacked sufficient country buy-in and, therefore, were unsustainable. A closer examination of OTSS model implementation in supported countries revealed signs of country ownership. The most notable was the tailoring of the OTSS approach and its implementation by countries. To varying degrees, countries tailored standard OTSS checklists to suit their specific needs and interests, although efforts were made to maintain a consistent set of core data and indicators across supported countries (e.g., percentage of patients testing negative for malaria prescribed malaria treatment). Countries, including Ghana and Zambia, also decentralized supervisory, management, and analytic functions progressively (facilitated by the EDS tool) to the district level, which also facilitated scale-up of OTSS visits to the national level.

By the end of the IMaD Project in 2012, Benin’s OTSS program had been spun-off and supported by a PMI-funded bilateral partner. At the close of MalariaCare in 2017, the National Malaria Control Program in Tanzania had taken over implementation of the OTSS model and the country’s EDS. It further adapted the approach in subsequent years, adding a data quality improvement component and rebranding the approach as the Malaria Service Delivery Quality Improvement program.^23^ The National Malaria Elimination Center in Zambia also took over management of the EDS tool and expanded the OTSS model nationally by dividing implementation support geographically between MalariaCare, the Promoting Advancement in Malaria Outcomes Project, and a Global Fund–supported program covering participating Churches Hospital Association of Zambia facilities. The Ghana Health Service, considering how this program would be managed appropriately in the long-term, assigned leadership of laboratory OTSS to its laboratory division, whereas clinical OTSS was led by the NMP. These early signs of country ownership, and adoption by other projects and donors, provided some hope that the OTSS model could be sustainable.

## THE U.S. PRESIDENT’S MALARIA INITIATIVE IMPACT MALARIA PROJECT AND THE OTSS PLUS APPROACH

After more than a decade of PMI support for the OTSS model and the larger QI package, and with additional published evidence that these efforts improved the quality of malaria diagnosis and case management,^21^^,^^24^^,^^25^ the PMI Impact Malaria Project launched in 2018 with a broader mandate. It would continue expansion of OTSS and related QI activities while adding support for QI of antenatal care (ANC) services to strengthen prevention and treatment of malaria in pregnancy (MIP). It also was expected to expand the collection and use of data to drive evidence-based decision making.

In the first years of PMI Impact Malaria, the OTSS model was launched in four new countries (Cameroon, Côte d’Ivoire, Niger, and Sierra Leone).^26^ In tandem with these launches, OTSS checklists were reviewed and streamlined through a consultative process with multiple country stakeholders, and harmonized to ensure a core set of indicators could be measured across countries. An ANC checklist to be administered by supervisory midwives was added to assess adherence to guidance on intermittent preventive treatment in pregnancy (IPTp) and provision of ITNs for pregnant women attending ANC. A separate checklist was developed for management of pregnant women with fever/suspected malaria. In addition, a record review checklist was developed to assess the management of severe malaria and its complications in inpatient facilities.

Rebranded as Outreach Training and Supportive Supervision Plus (OTSS+), this approach also increased focus on gender-sensitive and respectful care in the checklists and the training of supervisors. For example, health workers were assessed on whether they greeted the patient or caregiver, whether they informed the patient of the diagnosis and provided counseling, and whether they provided the opportunity for the patient or caregiver to ask questions.

This OTSS+ package was then presented to each MOH/NMP for review and adaptation, with the goal of having the MOH adopt the package formally for QI of health facilities. Seven countries, including the four new countries plus Madagascar, Malawi, and Mali, adopted the new OTSS+ package formally. Kenya and Zambia adopted components of the new package, and Rwanda adopted the ANC checklist as a companion to its existing clinical supervision checklists.

Beyond the updated and expanded checklist portfolio, PMI Impact Malaria brought further innovations into the OTSS+ package, including transitioning its primary data system from an EDS to the Health Network Quality Improvement System (HNQIS). The EDS tool had already demonstrated that digital data collection was feasible to implement and improved data quality, timeliness, and supervisors’ experiences. The HNQIS, an open-source, DHIS2-based software, brought with it a few additional features that changed significantly how data could be used.^27^ The HNQIS software included a built-in analytic capacity that would generate offline, real-time assessment scores for each checklist, as well as provide scores for each checklist section. This functionality enabled supervisors to share checklist scores with facility staff at the conclusion of the OTSS+ visit and to develop an action plan for addressing areas of poor performance. The HNQIS also added a feature that made it easier for supervisors to document and track action plans and progress through successive visits. Action plans help drive the QI process between OTSS+ visits, providing a framework for supervisors and health workers to discuss and monitor progress. Supervisors are encouraged to review action plans from previous visits before returning to a health facility, helping them set an agenda that prioritizes unresolved issues. The HNQIS was adopted quickly in those countries that launched the OTSS+ model with the support of the PMI Impact Malaria Project. Several countries, including Kenya, Malawi, and Zambia, which had been implementing the OTSS approach prior to the PMI Impact Malaria Project, also transitioned over time from using an EDS tool to the HNQIS to gain the improved functionalities of the new system.

Data collected through the HNQIS was uploaded automatically into the project’s data hub, which enabled PMI Impact Malaria country staff and their NMP counterparts to examine trends systematically over time in their countries and allowed project headquarters staff to conduct cross-country thematic analyses. When scores or trends were unsatisfactory or unexpected, subanalyses of the components of the checklist score were often conducted to determine whether there were specific components driving those low scores. For example, a subanalysis might have demonstrated that stock outages of commodities were a major factor resulting in low competency, or—alternatively—it might be the clinician’s failure to conduct a diagnostic test on suspected malaria cases. Armed with this information, supervisors and health authorities targeted follow-up actions to the specific areas of weakness.

**Figure 2.**
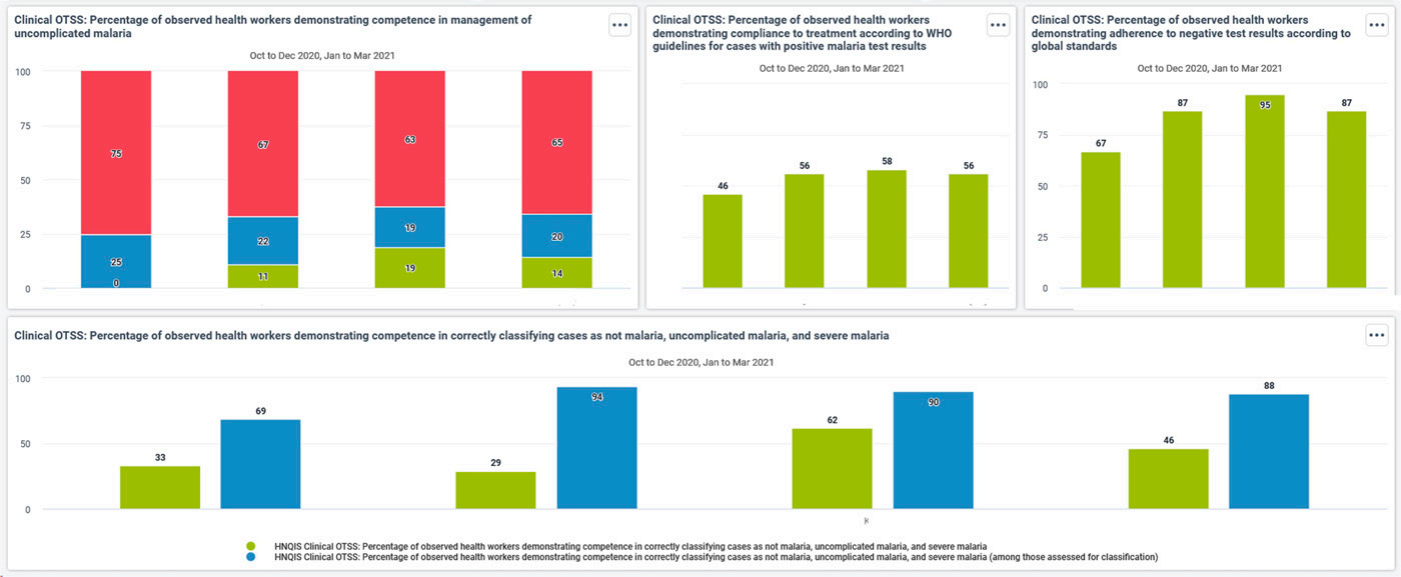
Example of an autogenerated data dashboard to display trends in scores for OTSS+ indicators. HNQIS = Health Network Quality Improvement System; OTSS+ = Outreach Training and Supportive Supervision Plus.

At the country level, the data collected through the HNQIS allowed the development of tailored data dashboards for use by project staff and NMPs at national, regional, and district levels that were accessible through a web-based, password-protected login page (Figure 2). These dashboards generated data tables and visualizations of key scores and indicators from the data hub automatically that could be used by MOH/NMP staff to assess progress in QI and to use those data in data review meetings. In many cases, this was the first time that these health authorities were equipped with real-time data on the quality of malaria service delivery to inform decision making about program management and targeting.

## A NEW QUALITY IMPROVEMENT FRAMEWORK

Despite the documented improvements in the quality of care in facilities supported by the OTSS+ model and the increasing scale of its implementation, MOH/NMP staff and other stakeholders also voiced some concerns. First among them were the substantial human and financial resources required to mount an OTSS+ round (A defined period of time during which a number of facilities are visited).

Although the OTSS model was originally envisioned to have a quarterly visit schedule, countries rarely had the logistical capacity, skilled human resources, or financing to visit each facility four times per year. Some countries have only managed to conduct yearly visits to the targeted facilities. Competing ITN and seasonal malaria chemoprevention (SMC) campaigns often led to delays in OTSS+ visits because key staff where taken away from their duties. Countries also often lack the financial resources and skilled human capacity to visit all facilities, and often identify and prioritize poorly performing facilities or only target selected geographic areas.

Some countries, including Cameroon, Côte d’Ivoire, and Kenya, sought to reduce the frequency of OTSS+ visits and to target support to lower performing facilities by adopting blended QI strategies that combined the OTSS+ model with complementary QI approaches, including mentorship, peer-to-peer learning, and virtual approaches. The data gathered from OTSS+ visits were often used to identify low-performing facilities, which would then be targeted for mentorship visits in between OTSS+ rounds. Mentors would use the results of previous OTSS+ visits to address specific areas of weakness. As mentored health workers improved their performance in subsequent OTSS+ visits, mentors could then move to other low-performing facilities. Deploying mentorship in this manner allowed countries to reduce the frequency of OTSS+ visits to once or twice yearly.

The COVID-19 pandemic and its resultant travel restrictions stimulated the development of creative approaches to sustain OTSS+ and other QI activities. The PMI Impact Malaria Project supported supervisors and mentors in developing WhatsApp groups to follow up on action plans, provide a forum for health workers to ask questions, and conduct virtual mentorship. In the Democratic Republic of the Congo, scheduled OTSS+ supervisor training was conducted through a blended approach, with regional trainers present in the classroom, and national and international trainers presenting through videoconferencing. Even after travel restrictions were lifted, countries have continued to use hybrid approaches to maintain contact and provide information to their supervisees.^27^

Another innovation deployed in all PMI Impact Malaria–supported countries focused on building district-level capacity to analyze and use data to monitor trends in key indicators, and to identify areas that required corrective actions. Using quarterly district data reviews, supervisors and facility heads shared OTSS+ and health management information system data with their peers, along with lessons learned and best practices on how to improve their performance. At the national level, lessons-learned workshops used a similar peer-to-peer learning approach, bringing together NMPs and key national stakeholders with district and regional health authorities to review the most recent round of OTSS+ visits and develop plans to address identified deficiencies.

The lessons learned from these country innovations resulted in the PMI Impact Malaria Project developing an updated QI framework, with the OTSS+ approach remaining the cornerstone of its QI efforts and its main source of QI data. Mentorship, peer-to-peer learning, and classroom training complement the OTSS+ model. Adoption of this QI framework spurred further innovations. Some components of supervisor training are currently being converted into an e-learning format. Additional e-learning courses on how to perform an mRDT, how to manage severe malaria and administer rectal artesunate, and how to assess for gestational age when deciding when to initiate IPTp also are under development.

## DIVERSIFICATION AND INSTITUTIONALIZATION

Although the COVID-19 pandemic presented major challenges to OTSS+ model implementation, it also presented opportunities to test the adaptability of the approach to expand its focus beyond malaria. In 2020, PMI Impact Malaria was requested to support facility-based QI for COVID-19 in Cameroon, the Democratic Republic of the Congo, and Ghana, where it already supported the OTSS+ model.^28^ The PMI Impact Malaria Project worked quickly with those countries to develop OTSS+ checklists on respiratory specimen collection and processing, biosafety, personal protection, clinical triage, and management of suspected COVID-19 cases, aligned with global and national policy guidance. The existing OTSS+ networks and supervisors in these countries provided the platform to implement these new checklists. In many cases, the COVID-19 supervision was conducted in tandem with planned malaria OTSS+ visits. Based on the initial implementation of the COVID-19 OTSS+ modules, both Cameroon and the Democratic Republic of the Congo have integrated these checklists into their ongoing routine OTSS+ visits.

The OTSS model also has served as the platform for countries to expand QI activities for a broader range of public health priorities. With PMI Impact Malaria support, the Ghana Health Service developed and launched integrated supportive supervision, which is used to monitor the performance of health facilities in multiple technical areas, including child and reproductive health and malaria, as part of the implementation of the country’s national health insurance scheme. Malawi is following suit and has plans to launch “integrated OTSS,” which expands the technical focus of OTSS to cover supply chain and data quality, similar to the approach used in Tanzania. These local adaptations are likely key to the success of sustaining the intervention.^29^

Early in the development of the OTSS model, it was recognized that long-term success required efforts to institutionalize this approach within country primary health-care systems. With that goal in mind, OTSS+ supervisors are drawn from district and regional health authorities and facilities. District and regional health offices were engaged in implementing OTSS+ visits, chaired data review meetings, and participated in developing and implementing action plans to address cross-cutting challenges. Ministry of Health staff led the review, adaptation, validation, and formal adoption of all checklists.

Learning lessons from prior projects, during which OTSS data were sometimes lost as one project closed and another was launched, PMI Impact Malaria has prioritized institutionalization of OTSS data systems, shifting data management from the project to MOHs in the Democratic Republic of the Congo, Tanzania, and Zambia, and to bilateral projects in Benin and Côte d’Ivoire. Data system transitioning also is underway in the remaining OTSS+ countries and is tailored to government systems and digital policies. To support this transition, the PMI Impact Malaria Project is purchasing country-based servers or securing cloud server capacity, training appropriate MOH staff in data systems administration, and building the capacity of relevant national and subnational health staff to analyze and use data.

Financing of the OTSS model also has evolved. In Ghana and Tanzania, support for OTSS visits and logistics is provided directly by the MOH, with the PMI providing funding support directly to the government. Although it is anticipated that ongoing technical assistance and financial support will continue to be a need in most countries implementing the OTSS model, in some countries, such as Ghana and Zambia, those needs are diminishing over time.

## NEW CHALLENGES AND OPPORTUNITIES FOR EXPANSION

During 5 years of implementation, the PMI Impact Malaria Project has supported NMPs to conduct 11,403 OTSS+ visits to 4,805 health facilities in 10 countries.^30^ These figures do not include OTSS visits conducted by other partners and countries, including Benin and Tanzania. A small number of countries have achieved national or near-national scale, including Ghana, Madagascar, and Zambia. Many other countries are still in the process of scaling up or have targeted the OTSS+ model specifically to limited geographic areas (Niger and Cameroon) or to specific facility types (the Democratic Republic of the Congo). In the absence of reliable information on the numbers of facilities in many targeted countries, accurate estimates of the coverage of the OTSS+ approach are elusive. Nonetheless, many countries have much more work to do to expand OTSS+ to all facilities that would benefit from it.

The main barrier to additional expansion of the OTSS+ model is financial. Countries and donors often prioritize support for preventive strategies (e.g., ITNs, SMC) over support for case management.^31^ An analysis conducted in 2017 during MalariaCare, examining cost data for daily allowances and transport (the main cost drivers for the OTSS model) for the last OTSS visit in seven countries, calculated a cost range of $44 to $333 per visit.^32^ Costs were higher when supervisors had to travel longer distances, stay overnight to complete the supervision visits, or had to hire vehicles for travel. With the OTSS+ model, in which supervisors are based at the district level and can complete an OTSS+ visit and return home in a single day, costs will lower per visit. Based on these data, if we were to construct a hypothetical scenario where a medium-size country with 5,000 health facilities received OTSS visits twice yearly at a cost of $250 per visit (taking into account recent global inflation), the cost for OTSS visits would be approximately $2.5 million per year. Of course, this is an oversimplification of visit costs and does not include the costs for additional activities, such as supervisor refresher training and review meetings. Nonetheless, the cost range of running an OTSS program at scale would be a small component of many countries’ current budgets for malaria control/elimination.

The lack of skilled human resources also is a barrier to achieving scale in many countries. The experiences through the IMaD, MalariaCare, and PMI Impact Malaria projects clearly demonstrate, though, that supervisory skills can be developed and strengthened at subnational levels, facilitating OTSS+ expansion.

In most countries, OTSS+ and complementary QI approaches have focused primarily on outpatient care at public health facilities. The PMI Impact Malaria Project developed an OTSS+ severe malaria checklist for inpatient units that relies on record reviews. Given the complexity of severe malaria and its management, this approach to QI has its limitations, because it can only assess a limited number of clinical scenarios and does not provide opportunities for supervisors to observe providers and offer coaching and on-the-job training. In Cameroon, Kenya, and Niger, PMI Impact Malaria has supported NMPs to pilot alternative approaches to improve inpatient management of severe malaria. These programs seek to build mentoring relationships between well-trained and experienced clinicians and their colleagues, using tools such as individual case reviews and targeted onsite training linked to actual clinical cases in those facilities. They also use virtual approaches such as virtual mentoring and WhatsApp group chats that allow clinicians to seek timely guidance from their peers. These pilots, which are ongoing, will determine whether such approaches are feasible and effective.

As countries have scaled up integrated community case management (iCCM) programs progressively, they have been challenged on how to improve and maintain the quality of services provided outside of fixed health facilities. The lack of proven QI strategies for iCCM and the challenges of supervising a large number of highly dispersed community health workers (CHWs), often based in remote settings, has impeded countries in these efforts. Building off the lessons learned through facility OTSS+, PMI Impact Malaria has developed a competency-based observation checklist that aligns with the standard iCCM algorithm. A companion readiness checklist examines record keeping and the availability of essential equipment and commodities. This community OTSS approach is being piloted in Cameroon, Mali, and Niger, with each country using different implementation approaches (Y. M. Bernard, personal communication). For example, Cameroon organizes so-called rally posts that bring together multiple CHWs in a single accessible location, similar to a health fair. This allows patients and caretakers access to needed services, and also enables supervisors to observe multiple CHWs managing sick children at the same time and location. Approaches like this can improve efficiency, but require planning, community mobilization, and coordination of key stakeholders.

Some countries also have expanded the OTSS+ approach to a limited number of private-sector facilities, primarily private hospitals and clinics. In these settings, the current OTSS+ approach can be implemented without major adaptations. Quality improvement for other segments of the private sector—particularly private pharmacies and medicine sellers, which are often a major source of malaria treatment—still faces major regulatory and logistical barriers that must be overcome if we are to achieve the goals of eliminating disease and death from malaria.^33^^,^^34^

## CONCLUSION

The continued commitment of the PMI to improving the quality of service delivery is a key component of its 2021 to 2026 strategy, End Malaria Faster, which builds on progress already made to expand access to quality services, reach the unreached, tailor interventions based on data, address threats to malaria programming by developing resilient malaria services, and take advantage of opportunities to end malaria within our lifetime.^35^

This supplement includes articles that demonstrate the effectiveness of the OTSS+ model and complementary QI approaches in improving the quality of clinical services for malaria. The articles in this supplement also document the feasibility of implementing this approach at scale in multiple countries by building supervisory, management, and analytic capacities at national and subnational levels. The OTSS+ model has also demonstrated flexibility in incorporating new QI objectives, such as MIP, data quality monitoring, and COVID-19, as well as expanding beyond outpatient care to incorporate community and inpatient services.

Through 15 years of investment in OTSS and other QI approaches, the PMI has supported a continuous cycle of implementation, learning, refinement, and expansion that has led to measurable improvements in malaria services and the health systems that support it. However, much more can and should be done to improve the access to and quality of malaria service delivery.

## Supplemental Materials

10.4269/ajtmh.23-0207 Supplemental Materials
