# Supplementary material for: The U.S. President’s Malaria Initiative’s Support for Improving the Quality of Malaria Case Management Services: Fifteen Years of Progress and Learning
Source: Am J Trop Med Hyg. 2023 Nov 27;110(3 Suppl):1–9. doi: 10.4269/ajtmh.23-0207 (PMC10919229; doi:10.4269/ajtmh.23-0207)
Supplement: Supplemental Materials [file tpmd230207.SD1.pdf]

Checklist: Assessing clinical management of clients suspected of having malaria

Health facility information

Name of Health Facility	
Type of Health Facility	
Number of health care workers responsible for malaria services	
Province/Region	
District	
Name of Head of Health Facility and Qualification	
Signature of Head of Health Facility	
Date of Visit (DD/MM/YYYY)	
Supervisor's Name	
Supervisor's Cadre	☐ Clinical ☐ Lab ☐ Other
Supervisor's Gender	☐ Female ☐ Male
Supervisor's Signature:	
Supervisor's Phone Number:	

	N°	Critical Questions	Response Options
Reception	1	Did the provider greet the client at the entrance of the consultation office?	☐ Yes<br>☐ No
	2	Did the provider invite the client to sit down?	☐ Yes<br>☐ No
	3	Did the provider introduce themself to the client?	☐ Yes<br>☐ No
	4	Did the provider treat the client with kindness and respect (Listening with attention, showing interest to the client as a person) throughout the visit?	☐ Yes<br>☐ No
Collecting historical client information	5	Did the provider ask the client's age?	☐ Yes<br>☐ No
	6	If the answer to #5 is Yes:<br>Is the client over 12 years old?	☐ Yes<br>☐ No
	7	If the answer to #6 is Yes:<br>Is the client a woman of childbearing age?	☐ Yes<br>☐ No
	8	Did the provider ask where the client lived?	☐ Yes<br>☐ No
	9	Did the provider look for a sign of fever or ask about a history of fever over the two previous days?	☐ Yes<br>☐ No
	10	Did the provider ask about the symptoms of uncomplicated malaria (fever, chills, sweating, headache, aches, nausea, lack of appetite, etc.)?	☐ Yes<br>☐ No
	11	Did the provider ask questions looking for signs of severe malaria in the client (convulsions, agitation, confusion, prostration, difficulty breathing, spontaneous bleeding, dark urine, etc.)?	☐ Yes<br>☐ No
	12	If the answer to #7 is Yes:<br>If a woman of childbearing age, has the health provider asked about pregnancy status or DDR?	☐ Yes<br>☐ No<br>☐ Not child-bearing age
	13	Did the provider ask if the client has taken any malaria treatment in the last two weeks?	☐ Yes<br>☐ No
	14	Did the health provider ask about any drugs already taken by the client?	☐ Yes<br>☐ No

Diagnosis	15	Did the provider take the temperature?	☐ Yes<br>☐ Done at consulting<br>☐ No
	16	Did the provider measure the client's weight?	☐ Yes<br>☐ Done at consulting<br>☐ No
	17	If the answer to #6 is Yes:<br>Did the provider take the client's blood pressure?	☐ Yes<br>☐ Done at consulting<br>☐ No
	18	Did the provider take the client's pulse?	☐ Yes<br>☐ Done at consulting<br>☐ No

**Diagnosis**	19	Did the provider take the client's respiratory rate?	☐ Yes<br>☐ Done at consulting<br>☐ No
	20	Did the provider look for signs of anemia in the client (conjunctivae, palms)?	☐ Yes<br>☐ No
	21	Did the provider look for signs of other serious illnesses (eyes, neck, etc.)?	☐ Yes<br>☐ No
	22	Did the provider perform a complete physical examination of the client (eyes, conjunctivae, fundus, palms, ears, throat, neck, heart, lungs, abdomen, and reflex)?	☐ Yes<br>☐ No
	23	Did the provider request a biological test to confirm malaria?	☐ Yes<br>☐ No

**Classification of malaria**	24	If the answer to #23 is Yes:<br>What was the result of the test?	☐ Positive<br>☐ Negative<br>☐ Inconclusive
	25	If the answer to #24 is Positive:<br>What type of malaria was categorized by the provider?	☐ Uncomplicated Malaria<br>☐ Severe Malaria<br>☐ No classification
	26	If the answer to #24 is Positive:<br>Does the supervisor agree with the provider's malaria classification?	☐ Yes<br>☐ No
	27	If the answer to #23 is Yes:<br>Did the provider explain the meaning of the result (positive or negative) of the test to the client and/or family and detail the appropriate course of action?	☐ Yes<br>☐ No
**Adherence to negative test**	28	If the answer to #24 is Negative:<br>Has the provider refrained from prescribing or administering an antimalarial drug to the client based on the NEGATIVE result of the malaria laboratory test (RDT or microscopy)?	☐ Yes<br>☐ No
**Treatment of uncomplicated malaria**	29	If the answer to #25 is Uncomplicated Malaria:<br>Is the client pregnant?	☐ Yes<br>☐ No
	30	If the answer to #29 is No:<br>Did the provider use a recommended ACT to treat uncomplicated malaria in the client as directed by national guidelines?	☐ Yes<br>☐ No
	31	If the answer to #29 is Yes:<br>If the client is pregnant, what is the gestational age?	☐ 1st trimester<br>☐ 2nd trimester<br>☐ 3rd trimester
	32	If the answer to #31 is 2nd or 3rd trimester:<br>Did the provider use an ACT recommended to treat uncomplicated malaria in clients including pregnant women in the 2nd and 3rd trimester of pregnancy as directed?	☐ Yes<br>☐ No

**Treatment of uncomplicated malaria**	33	If the answer to #32 is 1st trimester:<br>If the client is a pregnant woman in the first trimester of pregnancy, has the provider used quinine tablets to treat uncomplicated malaria according to national guidelines?	☐ Yes<br>☐ No
	34	If the answer to #25 is Uncomplicated Malaria:<br>Did the provider use the correct dosage to treat uncomplicated malaria?	☐ Yes<br>☐ No
	35	If the answer to #25 is Uncomplicated Malaria:<br>Is the dosage and duration of treatment explained to the client?	☐ Yes<br>☐ No
	36	If the answer to #35 is Yes:<br>Did the provider verify that the client understood the explanations given?	☐ Yes<br>☐ No
	37	If the answer to #25 is Uncomplicated Malaria:<br>Did the provider inform the client of the potential undesirable effects of the drug used?	☐ Yes<br>☐ No
	38	If the answer to #25 is Uncomplicated Malaria:<br>Did the provider invite the client back if he/she has complications?	☐ Yes<br>☐ No
	39	If the answer to #25 is Uncomplicated Malaria:<br>Did the provider give the client a follow-up appointment?	☐ Yes<br>☐ No

In Case of Transfer

**Pre-referral treatment for severe malaria**	40	If the answer to #25 is Severe Malaria:<br>Did the provider administer a pre-transfer treatment to the client?	☐ Yes<br>☐ No
	41	If the answer to #40 is Yes:<br>Which pre-transfer treatment did the provider administer to the client?	☐ Artesunate<br>☐ Artemether Injection
	42	If the answer to #41 is Artesunate:<br>If artesunate was used for the pre-transfer treatment, did the provider calculate the correct amount based on the client's weight?	☐ Yes<br>☐ No
	43 - 46	If the answer to #41 is Artesunate:<br>Did the provider follow the procedure for the preparation of the artesunate injection? *(i- Removed and injected the entire contents (1ml) of the sodium bicarbonate ampoule into the artesunate powder vial; ii- shook gently until dissolved and a clear reconstituted solution was obtained; iii- removed and injected the required volume of 5% saline or dextrose solution (depending on IV or IM) into the reconstituted artesunate solution; iv- removed the dose (in ml) required for IV or IM administration from the vial(s) prepared beforehand and injected the solution).*	☐ Yes<br>☐ No
	47	If the answer to #41 is Artesunate:<br>Did the provider administer a weight-appropriate dose of artesunate based on IV or IM route?	☐ Yes<br>☐ No
	48	If the answer to #41 is Artemether Injection:<br>If artemether injection is used for pre-transfer treatment, is the dosage as directed?	☐ Yes<br>☐ No
	49	If the answer to #25 is Severe Malaria:<br>Did the provider provide a reference note to the client's parents?	☐ Yes<br>☐ No

**Conclusion of visit**	50	Did the provider record all the information collected from the client in their register?	☐ Yes<br>☐ No
	51	Did the provider provide to the client the opportunity to ask question?	☐ Yes<br>☐ No
	52	Did the provider give the client and his/her family appropriate advice on malaria (causes, risks, prevention)?	☐ Yes<br>☐ No

Immediate Corrective Actions

Briefly describe key activities conducted during Supportive Supervision visit and number of staff reached

Action Taken		# of Staff Reached
1	**Category:** ☐ Lecture; ☐ Demonstration; ☐ Mentoring/Coaching ☐ Document/SOP development; ☐ Other	
	Describe:	
2	**Category:** ☐ Lecture; ☐ Demonstration; ☐ Mentoring/Coaching ☐ Document/SOP development; ☐ Other	
	Describe:	
3	**Category:** ☐ Lecture; ☐ Demonstration; ☐ Mentoring/Coaching ☐ Document/SOP development; ☐ Other	
	Describe:	
4	**Category:** ☐ Lecture; ☐ Demonstration; ☐ Mentoring/Coaching ☐ Document/SOP development; ☐ Other	
	Describe:	
5	**Category:** ☐ Lecture; ☐ Demonstration; ☐ Mentoring/Coaching ☐ Document/SOP development; ☐ Other	
	Describe:	
6	**Category:** ☐ Lecture; ☐ Demonstration; ☐ Mentoring/Coaching ☐ Document/SOP development; ☐ Other	
	Describe:	
Total	Total number of staff reached across all activities (Note: there should be no double-counting across categories for this total)	

Supervisor Feedback

	Observation 1	Observation 2	Observation 3
Supervisor: Did you provide feedback to staff on issues identified during observation for each aspect: Greeting, clinical history, diagnosis, treatment, admission, referral, and counseling?	☐ Yes<br>☐ No	☐ Yes<br>☐ No	☐ Yes<br>☐ No
If "No," why not?<br>(Write the reason number in the box for each observation)<br><br>1. No febrile patient<br>2. No clinician available<br>3. Patient referred during HF visit<br>4. Stopped consultation due to potential harm<br>5. Not enough time<br>6. Other (explain)	Reason #:_____<br><br>Explain:	Reason #:_____<br><br>Explain:	Reason #:_____<br><br>Explain:
Based on your overall observations, how would you rate this HF in terms of its general examination for malaria?	☐ Unsatisfactory<br>☐ Fair<br>☐ Satisfactory<br>☐ Excellent		

Additional comments on the observations

**Observation 1**
**Observation 2**
**Observation 3**

Recommendation and Action Plan

- If you found more than one gap (questions for which there is missing, partially complete, or incomplete answer or failure to follow national protocols) identified during the last HF visit, or if you have identified more than one during the current visit, make a copy of this page for each gap.
- Leave a copy of the supervisor feedback and action plan at the HF.
-

Date of HF visit:		Number of staff supervised:	M: ☐ F: ☐
What were the biggest gap(s) identified during the *last* HF visit?			
Briefly describe gap(s):		Briefly describe the action plan laid out in the previous visit:	
Briefly describe the action plan laid out in the previous visit:			
☐ Not addressed	☐ Partially addressed	☐ Completely addressed	
If addressed, explain the actions taken. If partially or not addressed, what is the new action plan to address the gap(s):			

What were the biggest gap(s) identified *today*?			
Briefly describe gap(s):	____<br>____<br>____<br>____	Was immediate feedback provided?	☐ Yes<br>☐ No
If yes, how was the feedback provided?			
☐ Guideline Review ☐ Demonstration ☐ SOP Review<br>☐ Clinical mentoring ☐ Lecture ☐ Other (specify): ______________			

Identified gap	Recommended action	Responsible person(s)	Timeline	Update/Status

3. Supervisor comments:

____
____
____
____
____

Checklist: Assessing competencies in MIP at ANC

Health facility information

Name of Health Facility	
Type of Health Facility	
Number of health care workers responsible for malaria services	
Province/Region	
District	
Name of Head of Health Facility and Qualification	
Signature of Head of Health Facility	
Date of Visit (DD/MM/YYYY)	
Supervisor's Name	
Supervisor's Cadre	☐ Clinical ☐ Lab ☐ Other
Supervisor's Gender	☐ Female ☐ Male
Supervisor's Signature:	
Supervisor's Phone Number:	

	N°	Critical steps	Response Options
**Reception**	1	Did the provider greet the client at the entrance of the consultation office?	☐ Yes<br>☐ No
	2	Did the provider invite the client to sit down?	☐ Yes<br>☐ No
	3	Did the provider introduce themself to the client?	☐ Yes<br>☐ No
	4	Did the provider treat the client with kindness and respect (Listening with attention, showing interest to the client as a person) throughout the visit?	☐ Yes<br>☐ No
**Collecting historical client information**	5	Did the provider ask the client's age?	☐ Yes<br>☐ No
	6	Did the provider ask where the client lived?	☐ Yes<br>☐ No
	7	Did the provider research the gestational period, date of last menstrual period, ultrasound, fundal height, ANC booklet?	☐ Yes<br>☐ No
	8	Did the provider ask if the client has taken any malaria treatment in the last two weeks?	☐ Yes<br>☐ No
	9	Did the provider ask about any drugs already taken by the client?	☐ Yes<br>☐ No
	10	Did the provider ask or investigate whether the client was at her first CPN?	☐ Yes<br>☐ No

**Malaria diagnosis**	11	Did the provider look for a sign of fever or ask about a history of fever during the previous 2 days?	☐ Yes<br>☐ No
	12	Did client have fever, signs of fever, or history of fever?	☐ Yes<br>☐ No
	13	If the answer to #11 is yes:<br>Did the provider ask about the symptoms of uncomplicated malaria (fever, chills, sweating, headache, aches, nausea, lack of appetite, etc.)?	☐ Yes<br>☐ No
	14	If the answer to #11 is yes:<br>Did the provider ask questions looking for signs of severe malaria (convulsions, agitation, confusion, prostration, etc.)?	☐ Yes<br>☐ No
	15	If the answer to #11 is yes:<br>Did the provider take the temperature?	☐ Yes<br>☐ No
	16	If the answer to #11 is yes:<br>Did the provider measure the client's weight?	☐ Yes<br>☐ No
	17	If the answer to #11 is yes:<br>Did the provider take the client's blood pressure?	☐ Yes<br>☐ No
	18	If the answer to #11 is yes:<br>Did the provider take the client's pulse?	☐ Yes<br>☐ No

**Malaria diagnosis**	19	If the answer to #11 is yes:<br>Did the provider take the client's respiratory rate?	☐ Yes<br>☐ No
	20	If the answer to #11 is yes:<br>Did the provider check for fetal heart sounds (2nd and 3rd trimester of pregnancy)?	☐ Yes<br>☐ No
	21	If the answer to #11 is yes:<br>If the result of the malaria test was positive, did the provider explain the result to the client?	☐ Yes<br>☐ No
	22	If the answer to #11 is yes:<br>Did the provider look for signs of anemia in the client (pallor of the conjunctivae/palms of hands)?	☐ Yes<br>☐ No
	23	If the answer to #11 is yes:<br>Did the provider look for signs of other serious illnesses (Urinary tract infections, hypertensive disorders etc.)?	☐ Yes<br>☐ No
	24	If the answer to #11 is yes:<br>Did the provider request a biological test to confirm malaria (RDT/Thick smear)?	☐ Yes<br>☐ No
	25	If the answer to #24 is Yes:<br>What was the result of the biological examination?	☐ Positive<br>☐ Negative<br>☐ Inconclusive
	26	If the answer to #25 is Positive:<br>If the result of the malaria test was positive, did the provider explain the result to the client?	☐ Yes<br>☐ No
	27	If the answer to #25 is Positive:<br>Did the provider categorize the severity of malaria according to national guidelines?	☐ Yes<br>☐ No
	28	If the answer to #27 is Yes:<br>What type of malaria was categorized by the health provider?	☐ Uncomplicated Malaria<br>☐ Severe Malaria<br>☐ No classification

**Antimalarial treatment**	29	If the answer to #28 is Uncomplicated Malaria:<br>If it is uncomplicated malaria, does the antimalarial drug used to treat the client comply with the guidelines?	☐ Yes<br>☐ No
	30	If the answer to #29 is Yes:<br>Did the provider use the correct dosage to treat uncomplicated malaria in pregnant women?	☐ Yes<br>☐ No
	31	If the answer to #28 is Uncomplicated Malaria:<br>Did the provider explain the dosage and duration of the drug to the client?	☐ Yes<br>☐ No
	32	If the answer to #31 is Yes:<br>Did the provider verify that the client understood the explanations given?	☐ Yes<br>☐ No
	33	If the answer to #28 is Uncomplicated Malaria:<br>Did the provider inform the client of the potential undesirable effects of the drug used?	☐ Yes<br>☐ No
	34	If the answer to #28 is Uncomplicated Malaria:<br>Did the provider invite the client to come back if she has complications?	☐ Yes<br>☐ No
	35	If the answer to #28 is Uncomplicated Malaria:<br>Did the provider give the client a follow-up appointment?	☐ Yes<br>☐ No

**Antimalarial treatment**	36	If the answer to #28 is Uncomplicated Malaria:<br>Did the provider enter information on the treatment into the register?	☐ Yes<br>☐ No
	37	If the answer to #28 is Severe Malaria:<br>If it is severe malaria, did the provider administer any pre-transfer treatment?	☐ Yes<br>☐ No
	38	If the answer to #37 is Yes:<br>Does the drug used for pre-transfer treatment of severe malaria in pregnant women comply with the guidelines?	☐ Yes<br>☐ No
	39	Did the provider record all the information collected from the client in the register?	☐ Yes<br>☐ No
	40	Has the client and/or her family received appropriate malaria counseling?	☐ Yes<br>☐ No
	41	Did the provider verify that the client understood the advice?	☐ Yes<br>☐ No

	N°	Critical Steps	Response Options
**Dispensing IPTp**	**42**	If the answer to #11 is No:<br>Or if the answer to #25 is Negative or Inconclusive:<br>Did the provider ask or investigate whether the client had taken SP in IPT during the previous four weeks?	☐ Yes<br>☐ No
	43	If the answer to #11 is No:<br>Or if the answer to #25 is Negative or Inconclusive:<br>Did the provider check (mother-child record) whether the client was on co-trimoxazole for HIV chemoprevention?	☐ Yes<br>☐ No
	44	If the answer to #11 is No:<br>Or if the answer to #25 is Negative or Inconclusive:<br>Did the provider communicate with the client about the consequences of malaria in pregnancy?	☐ Yes<br>☐ No
	45	If the answer to #11 is No:<br>Or if the answer to #25 is Negative or Inconclusive:<br>Did the provider make the client aware of the benefits of preventing malaria with SP?	☐ Yes<br>☐ No
	46	If the answer to #11 is No:<br>Or if the answer to #25 is Negative or Inconclusive:<br>Did the provider educate the client about the need to take at least three doses of SP before giving birth?	☐ Yes<br>☐ No
	47	If the answer to #11 is No:<br>Or if the answer to #25 is Negative or Inconclusive:<br>Did the provider verify whether the client is eligible for IPT during this ANC?	☐ Yes<br>☐ No
	48	If the answer to #47 is Yes:<br>Is the client eligible according to the above criteria?	☐ Yes<br>☐ No
	49	If the answer to #47 is No:<br>If not eligible, specify why	☐ Yes<br>☐ No
	50	If the answer to #11 is No:<br>Or if the answer to #25 is Negative or Inconclusive:<br>Did the provider inform the client about the potential side effects of SP?	☐ Yes<br>☐ No
	51	If the answer to #11 is No:<br>Or if the answer to #25 is Negative or Inconclusive:<br>Did the provider give three SP tablets to the client eligible for IPT?	☐ Yes<br>☐ No

**Dispensing**	52	If the answer to #51 is Yes:<br>Did the provider ask the client to drink water to swallow the three SP tablets on the spot?	☐ Yes<br>☐ No
	53	If the answer to #51 is Yes:<br>Did the provider observe the client taking the three SP tablets?	☐ Yes<br>☐ No
	54	If the answer to #11 is No:<br>Or if the answer to #25 is Negative or Inconclusive:<br>Did the provider encourage the client to return each month for IPT up until delivery?	☐ Yes<br>☐ No
	55	If the answer to #51 is Yes:<br>Did the provider ask the client to return to see them if there are any serious skin and/or digestive symptoms as a result of taking the SP?	☐ Yes<br>☐ No
	56	If the answer to #11 is No:<br>Or if the answer to #25 is Negative or Inconclusive:<br>Did the provider inform the client of the date of the next appointment?	☐ Yes<br>☐ No
	57	If the answer to #51 is Yes:<br>Did the provider record the dose of SP in the mother-child record and ANC registry in compliance with the national guidelines?	☐ Yes<br>☐ No
**LLIN distribution**	58	Did the provider explain the benefits of sleeping under an LLIN every night to prevent malaria?	☐ Yes<br>☐ No
	59	Did the pregnant client receive an LLIN for this pregnancy during the previous visits?	☐ Yes<br>☐ No
	60	If the answer to #59 is No:<br>Did the provider give the client an LLIN if she had not yet received it during this pregnancy?	☐ Yes<br>☐ No
	61	If the answer to #60 is Yes:<br>Did the provider explain to the client how to use the LLIN?	☐ Yes<br>☐ No
	62	If the answer to #60 is Yes:<br>Did the provider advise the client to sleep under LLINs during pregnancy and after delivery?	☐ Yes<br>☐ No
	63	If the answer to #60 is Yes:<br>Did the provider record the provision of the LLIN to the client in the mother-child record and in the ANC register?	☐ Yes<br>☐ No

**Dispensing anti-anemia drugs**	64	Did the provider give the client iron tablets after the consultation?	☐ Yes<br>☐ No
	65	Did the provider give the client 0.4 mg folic acid tablets after the consultation?	☐ Yes<br>☐ No
	66	If the answer to #65 is Yes:<br>Did the provider check that the client understood how to take the iron and folic acid tablets?	☐ Yes<br>☐ No
**Conclusion of visit**	67	Did the health provider provide the opportunity to the client to ask questions?	☐ Yes<br>☐ No
	68	Did the provider provide the client and her family appropriate advice on malaria (causes, risks, prevention)?	☐ Yes<br>☐ No

Immediate Corrective Actions

Briefly describe key activities conducted during Supportive Supervision visit and number of staff reached

	Action Taken	# of Staff Reached
1	**Category:** ☐ Lecture; ☐ Demonstration; ☐ Mentoring/Coaching ☐ Document/SOP development; ☐ Other	
	Describe:	
2	**Category:** ☐ Lecture; ☐ Demonstration; ☐ Mentoring/Coaching ☐ Document/SOP development; ☐ Other	
	Describe:	
3	**Category:** ☐ Lecture; ☐ Demonstration; ☐ Mentoring/Coaching ☐ Document/SOP development; ☐ Other	
	Describe:	
4	**Category:** ☐ Lecture; ☐ Demonstration; ☐ Mentoring/Coaching ☐ Document/SOP development; ☐ Other	
	Describe:	
5	**Category:** ☐ Lecture; ☐ Demonstration; ☐ Mentoring/Coaching ☐ Document/SOP development; ☐ Other	
	Describe:	
6	**Category:** ☐ Lecture; ☐ Demonstration; ☐ Mentoring/Coaching ☐ Document/SOP development; ☐ Other	
	Describe:	
Total	Total number of staff reached across all activities (Note: there should be no double-counting across categories for this total)	

Supervisor Feedback

	Observation 1	Observation 2	Observation 3
Supervisor: Did you provide feedback to staff on issues identified during observation for each aspect: Greeting, clinical history, diagnosis, treatment, admission, referral, and counseling?	☐ Yes<br>☐ No	☐ Yes<br>☐ No	☐ Yes<br>☐ No
If "No," why not?<br>(Write the reason number in the box for each observation)<br><br>1. No febrile patient<br>2. No clinician available<br>3. Patient referred during HF visit<br>4. Stopped consultation due to potential harm<br>5. Not enough time<br>6. Other (explain)	Reason #:_____<br><br>Explain:	Reason #:_____<br><br>Explain:	Reason #:_____<br><br>Explain:
Based on your overall observations, how would you rate this HF in terms of its general examination for malaria?	☐ Unsatisfactory<br>☐ Fair<br>☐ Satisfactory<br>☐ Excellent		

Additional comments on the observations

**Observation 1**
**Observation 2**
**Observation 3**

Recommendation and Action Plan

- If you found more than one gap (questions for which there is missing, partially complete, or incomplete answer or failure to follow national protocols) identified during the last HF visit, or if you have identified more than one during the current visit, make a copy of this page for each gap.
- Leave a copy of the supervisor feedback and action plan at the HF.
-

Date of HF visit:		Number of staff supervised:	M: F:
What were the biggest gap(s) identified during the *last* HF visit?			
Briefly describe gap(s):		Briefly describe the action plan laid out in the previous visit:	
Briefly describe the action plan laid out in the previous visit:			
☐ Not addressed	☐ Partially addressed	☐ Completely addressed	
If addressed, explain the actions taken. If partially or not addressed, what is the new action plan to address the gap(s):			

What were the biggest gap(s) identified *today*?			
Briefly describe gap(s):	____________<br>____________<br>____________<br>____________	Was immediate feedback provided?	☐ Yes<br>☐ No
If yes, how was the feedback provided?			
☐ Guideline Review ☐ Demonstration ☐ SOP Review<br>☐ Clinical mentoring ☐ Lecture ☐ Other (specify): _______________			

Identified gap	Recommended action	Responsible person(s)	Timeline	Update/Status

3. Supervisor comments:

____

____

____

____

____

Checklist: Assessing health facility readiness

Health facility information

Name of Health Facility	
Type of Health Facility	
Number of health care workers responsible for malaria services	
Province/Region	
District	
Name of Head of Health Facility and Qualification	
Signature of Head of Health Facility	
Date of Visit (DD/MM/YYYY)	
Supervisor's Name	
Supervisor's Cadre	☐ Clinical ☐ Lab ☐ Other
Supervisor's Gender	☐ Female ☐ Male
Supervisor's Signature:	
Supervisor's Phone Number:	

	N°	Critical questions	Response Options
Information (non-scored questions)	1	Does facility offer ANC services?	☐ Yes<br>☐ No
	2	Does facility distribute LLINs to children under 5 years of age?	☐ Yes<br>☐ No
	3	Does facility offer laboratory services?	☐ Yes<br>☐ No
	4	Does facility offer diazepam?	☐ Yes<br>☐ No
	5	Does facility offer blood transfusion?	☐ Yes<br>☐ No
	6	Is there at least 1 CHW attached to this facility?	☐ Yes<br>☐ No
	7	Does facility offer case management of severe malaria services?	☐ Yes<br>☐ No
		<u>Are these medications available today?</u>	
Availability of medications	8	Paracetamol	☐ Yes<br>☐ No
	9	If the answer to #1 is Yes:<br>Sulfadoxine-Pyrimethamine	☐ Yes<br>☐ No
	10	ACTs for children under 5 years	☐ Yes<br>☐ No
	11	ACTs for children 5-7 years	☐ Yes<br>☐ No
	12	ACTs for children 7-13 years	☐ Yes<br>☐ No
	13	ACTs for children over 13 and adults	☐ Yes<br>☐ No
	14	Injectable Quinine	☐ Yes<br>☐ No
	15	Compressed Quinine	☐ Yes<br>☐ No
	16	Injectable Artesunate and/or IM Injectable Artemether	☐ Yes<br>☐ No
	17	Artesunate Rectocaps	☐ Yes<br>☐ No
	18	If the answer to #1 is Yes:<br>Iron Tablets	☐ Yes<br>☐ No
	19	If the answer to #1 is Yes:<br>0.4 mg Folic Acid	☐ Yes<br>☐ No
	20	If the answer to #4 is Yes:<br>Diazepam	☐ Yes<br>☐ No
	21	5% Glucose	☐ Yes<br>☐ No
	22	10% Glucose	☐ Yes<br>☐ No

	23	30% Glucose	☐ Yes<br>☐ No
	24	Lactate Ringer	☐ Yes<br>☐ No
	25	Saline Solution	☐ Yes<br>☐ No

	N°	Critical Questions	Response Options
Availability of Commodities		Are these commodities available today?	
	26	Oxygen	☐ Yes<br>☐ No
	27	If the answer to #1 is Yes:<br>Drinking water at the site of ANC	☐ Yes<br>☐ No
	28	If the answer to #1 is Yes:<br>Disposable glasses available (or consistent cleaning) for the DOT strategy of the SP during ANC	☐ Yes<br>☐ No
	29	Are examination gloves available?	☐ Yes<br>☐ No
	30	Soap or hand sanitizer gel	☐ Yes<br>☐ No
	31	Sharps containers	☐ Yes<br>☐ No
	32	Thermometer	☐ Yes<br>☐ No
	33	Personal scale	☐ Yes<br>☐ No
	34	Syringes	☐ Yes<br>☐ No
	35	Alcohol	☐ Yes<br>☐ No
	36	Cotton	☐ Yes<br>☐ No
	37	Malaria RDTs	☐ Yes<br>☐ No
	38	If the answer to #1 is Yes:<br>LLINs	☐ Yes<br>☐ No
	39	If the answer to #3 is Yes:<br>Reagents for determining blood groups	☐ Yes<br>☐ No
	40	If the answer to #5 is Yes:<br>Blood for transfusion	☐ Yes<br>☐ No

**Availability of Documentation**		Are these documents available today?	
	41	Records of curative consultations	☐ Yes<br>☐ No
	42	If the answer to #1 is Yes:<br>Records of prenatal consultations	☐ Yes<br>☐ No
	43	If the answer to #2 is Yes:<br>Records of the distribution of LLINs to children under 5 years of age	☐ Yes<br>☐ No
	44	If the answer to #1 is Yes:<br>Records of the distribution of LLINs to pregnant women	☐ Yes<br>☐ No
	45	HMIS reports	☐ Yes<br>☐ No
	46	If the answer to #6 is Yes:<br>Monthly CHW reports	☐ Yes<br>☐ No
	47	Manual for filling out the data collection media	☐ Yes<br>☐ No
**Availability of Materials**		Are these materials (guides, job aides, etc) available today?	
	48	If the answer to #1 is Yes:<br>Malaria and pregnancy guide or tools/support on ANC	☐ Yes<br>☐ No
	49	If the answer to #1 is Yes:<br>ANC guidelines	☐ Yes<br>☐ No
	50	National guidelines for the management of malaria	☐ Yes<br>☐ No
	51	Management of uncomplicated malaria fact sheet	☐ Yes<br>☐ No
	52	If the answer to #7 is Yes:<br>Management of severe malaria fact sheet	☐ Yes<br>☐ No
	53	Management of malaria in pregnancy fact sheet	☐ Yes<br>☐ No
	54	Preparation of artesunate injection PEC fact sheet	☐ Yes<br>☐ No
	55	If the answer to #1 is Yes:<br>LLIN distribution guidelines	☐ Yes<br>☐ No

Availability of Trained Human Resources	N°	Critical Question	Response Options
	56	Physicians - Male	
	57	Physicians - Female	
	58	Midwives - Male	
	59	Midwives - Female	
	60	Nurses - Male	
	61	Nurses - Female	
	62	Pharmacists/PGP - Male	
	63	Pharmacists/PGP - Female	
	64	Does the health facility have at least 50% of staff trained to manage malaria cases using the latest national guidelines?	☐ Yes<br>☐ No

Availability of quality data	N°	Performance of the facility visited	Scores	
	1	If the answer to #1 is Yes:<br>Number of pregnant women received in ANC 1	Amount reported (R)	
			Amount found in the register (F)	
			Difference (F-R) = 0?	
	2	If the answer to #1 is Yes:<br>Number of pregnant women who received IPT 3	Amount reported (R)	
			Amount found in the register (F)	
			Difference (F-R) = 0?	
	3	Number of suspected malaria cases registered in the health facility	Amount reported (R)	
			Amount found in the register (F)	
			Difference (F-R) = 0?	
	4	Number of suspected malaria cases tested by RDT or thick smear test Luis B: If PMP includes TPR, we will need to capture N of tests +	Amount reported (R)	
			Amount found in the register (F)	
			Difference (F-R) = 0?	
	5	Number of uncomplicated malaria cases tested by RDT or thick smear test	Amount reported (R)	
			Amount found in the register (F)	
			Difference (F-R) = 0?	
	6	Number of confirmed uncomplicated malaria cases treated with ACTs	Amount reported (R)	
			Amount found in the register (F)	
			Difference (F-R) = 0?	
	7	Number of ACT treatments consumed (taken from the health facility's pharmacy)	Amount reported (R)	
			A mount found in the register (F)	
			Difference (F-R) = 0?	

Immediate Corrective Actions

Briefly describe key activities conducted during Supportive Supervision visit and number of staff reached

Action Taken		# of Staff Reached
1	**Category:** ☐ Lecture; ☐ Demonstration; ☐ Mentoring/Coaching ☐ Document/SOP development; ☐ Other	
	Describe:	
2	**Category:** ☐ Lecture; ☐ Demonstration; ☐ Mentoring/Coaching ☐ Document/SOP development; ☐ Other	
	Describe:	
3	**Category:** ☐ Lecture; ☐ Demonstration; ☐ Mentoring/Coaching ☐ Document/SOP development; ☐ Other	
	Describe:	
4	**Category:** ☐ Lecture; ☐ Demonstration; ☐ Mentoring/Coaching ☐ Document/SOP development; ☐ Other	
	Describe:	
5	**Category:** ☐ Lecture; ☐ Demonstration; ☐ Mentoring/Coaching ☐ Document/SOP development; ☐ Other	
	Describe:	
6	**Category:** ☐ Lecture; ☐ Demonstration; ☐ Mentoring/Coaching ☐ Document/SOP development; ☐ Other	
	Describe:	
Total	Total number of staff reached across all activities (Note: there should be no double-counting across categories for this total)	

Supervisor Feedback

	Observation 1	Observation 2	Observation 3
Supervisor: Did you provide feedback to staff on issues identified during observation for each aspect: Greeting, clinical history, diagnosis, treatment, admission, referral, and counseling?	☐ Yes<br>☐ No	☐ Yes<br>☐ No	☐ Yes<br>☐ No
If "No," why not?<br>(Write the reason number in the box for each observation)<br><br>1. No febrile patient<br>2. No clinician available<br>3. Patient referred during HF visit<br>4. Stopped consultation due to potential harm<br>5. Not enough time<br>6. Other (explain)	Reason #:____<br><br>Explain:	Reason #:____<br><br>Explain:	Reason #:____<br><br>Explain:
Based on your overall observations, how would you rate this HF in terms of its general examination for malaria?	☐ Unsatisfactory<br>☐ Fair<br>☐ Satisfactory<br>☐ Excellent		

Additional comments on the observations

**Observation 1**
**Observation 2**
**Observation 3**

Recommendation and Action Plan

- If you found more than one gap (questions for which there is missing, partially complete, or incomplete answer or failure to follow national protocols) identified during the last HF visit, or if you have identified more than one during the current visit, make a copy of this page for each gap.
- Leave a copy of the supervisor feedback and action plan at the HF.
-

Date of HF visit: ☐		Number of staff supervised:	M: ☐ F: ☐
What were the biggest gap(s) identified during the *last* HF visit?			
Briefly describe gap(s):		Briefly describe the action plan laid out in the previous visit:	
Briefly describe the action plan laid out in the previous visit:			
☐ Not addressed	☐ Partially addressed	☐ Completely addressed	
If addressed, explain the actions taken. If partially or not addressed, what is the new action plan to address the gap(s):<br>__________<br>__________<br>__________<br>__________			

What were the biggest gap(s) identified *today*?			
Briefly describe gap(s):	____________<br>____________<br>____________<br>____________	Was immediate feedback provided?	☐ Yes<br>☐ No
If yes, how was the feedback provided?			
☐ Guideline Review ☐ Demonstration ☐ SOP Review<br>☐ Clinical mentoring ☐ Lecture ☐ Other (specify): ____________			

Identified gap	Recommended action	Responsible person(s)	Timeline	Update/Status

3. Supervisor comments:

____

____

____

____

____
